# Analysis of Longevity Traits in Holstein Cattle: A Review

**DOI:** 10.3389/fgene.2021.695543

**Published:** 2021-08-03

**Authors:** Honghong Hu, Tong Mu, Yanfen Ma, XingPing Wang, Yun Ma

**Affiliations:** Ningxia Key Laboratory of Ruminant Molecular and Cellular Breeding, School of Agriculture, Ningxia University, Yinchuan, China

**Keywords:** Holstein cattle, longevity, heritability, breeding method, genetic correlation

## Abstract

Dairy cow longevity is an essential economic trait that can supplement the breeding value of production traits, which is related to the herd time and lifetime milk yield of dairy cows. However, longevity is a relatively difficult trait to select for dairy cow breeding due to low heritability and numerous influence factors of the longevity in dairy cows. Longevity trait has been used as an important breeding target of a comprehensive selection index in many dairy developed countries; however, it has not been included in performance index in many developing countries. At present, cows in these countries are still in the primary stage of “large quantity, low quality, high cost, and low yield.” The average parity of dairy cows is less than 2.7, which is difficult to maintain the production efficiency to meet the demands of the dairy industry. Therefore, there is an urgent need to select and breed for the longevity of dairy cows. The various definitions and models (including linear, threshold, random regression, sire, and survival analysis) of longevity were reviewed and standardized. Survival analysis is the optimal model to evaluate longevity, and the longevity heritability is 0.01–0.30 by using different definitions and models. Additionally, the relationship between longevity and other traits was summarized, and found that longevity was regulated by multiple factors, and there were low or medium genetic correlations between them. Conformation traits, milk production traits, reproductive traits, and health traits may be used as indicators to select and breed the longevity of dairy cows. The genetic assessment methods, heritability, influencing factors, importance, breeding, and genetics of longevity were reviewed in the manuscript, which could provide a valuable reference for the selective breeding to extend the productive life of Holstein cattle.

## Introduction

The longevity of dairy cows refers to the time from the first calving to exit the herd when cows do not have sufficient productivity. The production life of dairy cows is less than 3–4.5 years (Kerslake et al., 2018), but in fact the maximum annual production occur in the fifth lactation period, and the highest annual profit typically achieved in the sixth lactation period (Horn et al., 2012). The natural lifespan of cows is approximately 20 years, but the average culled time is much earlier than the natural life. Moreover, cows will be eliminated if they cannot reach the peak production to obtain the highest profit (Najafabadi et al., 2016).

The longevity of dairy cows is a complex trait with low heritability and a lack of supporting data, and longevity is affect by many factors, such as the inherent factors (lactation, health, conformation traits, and reproductive performance; Ferris et al., 2014) and the external factors (milk price, nutrition, management, policy, feeding cost, and replacement heifers; Grandl et al., 2016; Vries and Marcondes, 2020). Therefore, it is a difficult task for breeding longevity traits in dairy cows, and it is necessary to select the traits of longevity, which determines the utilization value of dairy cows, improves the economic benefits of dairy farms. It is especially important for the development of dairy industry.

Longevity of dairy cows was studied at home and abroad, and various definitions and methods of longevity were proposed (Table 1). However, because these terms are often used interchangeably and confusedly, which can confound the study of longevity traits. Therefore, it is necessary to standardize the terms of longevity traits. Herd life refers to the days from birth to culling or death (Zhang et al., 2021b), productive life refers to the days from the first calving to culling or death (Raguz et al., 2011), milking life refers to the days from the first calving to culling or death but excludes all dry periods (Zhang et al., 2021b), and stayability refers to the probability that a cow remains in the herd enough time to raise a certain number of calves that pay for her development and maintenance costs (Costa et al., 2020). Nevertheless, longevity is included as an important indicator in the comprehensive selection index of dairy cows in various countries. Prolonging the productive life of dairy cows can reduce involuntary culling (Zavadilová and Stipkova, 2012) and improve the voluntary culling of dairy cows (Sewalem et al., 2008), but can also help to increase the market profit of dairy enterprises (Albert, 2020), meet consumer demand, enhance animal welfare, respond to climate change, and promote environmentally sustainable development (Grandl et al., 2019). We mainly focuses on genetic evaluation, influencing factors, breeding, genetics, and breeding methods of longevity were reviewed to provide a reference for the selection and breeding of longevity traits in Holstein cattle.

**Table 1:** Terms and definitions of longevity in dairy cows in the literature.

Terms	Definitions	Source
Productive life	The day from first calving to culling.	Raguz et al., 2011
	The actual productive life and mainly depends on productivity.	Vukasinovic et al., 1997
	The total number of days in milk up to 84 mo of age with a restriction of 305, 500, or 999 d per lactation (PL305, PL500, or PL999, respectively).	Tsuruta et al., 2005
Functional productive life	The ability of the cow to avoid culling for involuntary reasons such as sterility or disease.	Vukasinovic et al., 1997
Length of productive life	The number of days from first calving until culling or censoring.	Caraviello et al., 2004
	The days between first calving and disposal.	Martinez et al., 2004
	The number of completed lactations.	Yazdi et al., 1999
Herd life	The day from first calving to culling.	Brickell and Wathes, 2011
	The total number of days from the first calving date to the last (culling) date.	Tsuruta et al., 2005
	The length of time that individual cows remain in herds after their first calving.	Hare et al., 2006
	Survival to the next lactation.	Jairath et al., 1998
Longevity	The total months in milk by 84 mo of age.	Vanraden and Klaaskate, 1993
	The number of days from first calving until culling or censoring	Sasaki et al., 2012
	The length of time during which an animal is able to stay producing in the herd, and survival.	Jamrozik et al., 2013
	The individual farmer to eliminate cows for low milk production (voluntary culling)	Strapáková et al., 2019
True longevity	The ability to delay any culling.	Jenko et al., 2013
Stayability	A measure of cow survival that does not require recording of cull data.	Handcock et al., 2020
	The probability of a cow remain in the herd enough time to raise a certain number of calves that pay for her development and maintenance costs.	Costa et al., 2020
	The ability of the cow to calve at least three times until 76 months of age.	Ramos et al., 2020
	The measure of whether or not an animal remains and produces in the herd until a specified point in time.	Jamrozik et al., 2013
Functional longevity	The number of days between the first calving and culling.	Zavadilova et al., 2011
	The different lengths for the time interval for survival.	Pelt et al., 2015
	The cow’s ability to avoid involuntary culling or culling not correlated with its own production.	Stanojević et al., 2018
	The capability of cows to delay involuntary culling for infertility or diseases.	Strapáková et al., 2019
Lifespan	The number of lactations an animal completes or is expected to complete prior to culling.	Brotherstone et al., 1997
Milking life	The days from the first calving to culling or death but excludes all dry periods.	Zhang et al., 2021b

## Genetic Evaluation and Heritability

Longevity is a very important economic trait. Several countries have explored different models to evaluate longevity based on different definitions, data properties, and quality to improve the selection of longevity traits by genetic evaluation. Typically, these models include linear models (Allaire, 1976), threshold models (Boettcher et al., 1999), random regression models (Pelt et al., 2015), sire models (Brotherstone et al., 1997), and survival analysis (Buenger et al., 2001). Linear models, threshold models, and random regression models can process multiple traits simultaneously; thereby directly estimate the genetic correlation between longevity and other traits with a relatively fast calculation speed (Imbayarwo-Chikosi et al., 2015). Survival analysis can appropriately accommodate censored data, consider time-dependent environmental impact, and manage the skewed distribution of longevity characteristics (Imbayarwo-Chikosi et al., 2016). The estimated value of the trait is remarkably close to the measured value, which can easily be adapted to longevity data and provide accurate results, but the calculation speed is relatively slow. In addition, linear, threshold models, and random regression models generally produce lower estimation of longevity heritability than survival analysis models on the original scale (Ducrocq, 1997; Setati et al., 2004; Jamrozik et al., 2008; Kern et al., 2014).

Survival analyses included parametric, semi-parametric, and non-parametric methods (Smith and Westgarth, 1957). Cox proportional hazard model is a semi-parametric method, and the Weibull distribution model is a parametric method, which have been used to estimate the longevity traits of dairy cows (Zhao, 2013). The Cox proportional hazard model is used to analyze the factors that affect the survival time without a clear benchmark risk rate function, which has a wide range of applications and shows high statistical efficiency (Stokes, 2019). The Weibull regression model is a multi-factor analysis model and is based on the Weibull distribution. The weight of each factor in the production life can be obtained with the change of time. The Weibull model can more adapt to the censoring, covariates changes with time, and the screening process more intuitively. Therefore, Weibull regression is more accurate than the Cox proportional hazard model, but it is also more complex.

Researchers use different models to genetically assess different longevity definitions, and find that the longevity heritability is low with rates 0.01–0.30 (Table 2). Although the selection process might be slow, there is sufficient genetic variation. The breeding of longevity traits could be improved indirectly by selecting traits that should be a strong genetic correlation with longevity. Indirect selection is useful if the square of the genetic correlation between the indirect trait and longevity is greater than the heritability of the longevity trait. In addition, the breeding of longevity traits could be improved by combining conventional breeding strategies with modern genome selection technology.

**Table 2:** Heritability of longevity.

Sample	Country	Trait	Model	Heritability	References
Holstein	America	Herd life	Linear mixed model	0.25	Allaire, 1976
Holstein	United Kingdom	Length of productive life	Sire	0.10	Hoque and Hodges, 1980
Holstein	America	Herd life	Sire	0.035	Dentine et al., 1987
Holstein	America	Herd life/Functional Herd life	Sire	0.03	Boldman et al., 1992
Holsteins	America	Herd life	Multiple – trait mixed model	0.01–0.10	Short and Lawlor, 1992
Holsteins	America	longevity	Linear model	0.085	Vanraden and Klaaskate, 1993
Swiss Brown	Switzerland	Herd life	Sire	0.03–0.14	Vukašinović et al., 1995
Black and White	Netherlands	Length of productive life/Herd life	Sire	0.035–0.136	Vollema and Groen, 1996
Holstein-Friesian dairy cattle	UK	Lifespan	Sire	0.063	Brotherstone et al., 1997
Cow	Germany	Functional longevity	Survival analysis	0.18	Buenger et al., 2001
Holsteins	America	Herd life/productive life	Multiple trait sire model	0.08–0.10	Tsuruta et al., 2005
South African Angus	South Africa	Stayability	Threshold	0.24–0.30	Maiwashe et al., 2009
Holstein	Tunisie	Longevity	Proportional hazard model	0.19	M’hamdi et al., 2010
Holstein	Japan	Longevity	Weibull proportional hazard model	0.119–0.123	Sasaki et al., 2012
Brown cattle	Slovenia	True longevity	Piecewise Weibull baseline Survival models	0.094	Jenko et al., 2013
		Functional longevity		0.099	
Black and white	Netherlands	Longevity	Random regression sire-maternal grandsire	0.115–0.149	
Dairy cattle	Dutch	Functional longevity	Random regression	0.146	Pelt et al., 2015
Holstein dairy	Iran	Length of productive life	Weibull proportional risk model	0.018	Najafabadi et al., 2016
Danish dairy cattle	Danish	Longevity	NAV longevity model	0.022–0.090	Clasen et al., 2017
South African Holstein Cattle	South Africa	Functional longevity	Weibull proportional hazards model	0.11	Imbayarwo-Chikosi et al., 2017
Holstein	Iran	Length of productive life/Functional productive life	Regression	0.032/0.012	Mirhabibi et al., 2018
Holstein	Slovak	Length of productive life	Weibull sire model	0.13	Strapáková et al., 2019
Slovak Simmental				0.05	

## Longevity with Other Traits

### Longevity and Conformation

**Figure 1:**
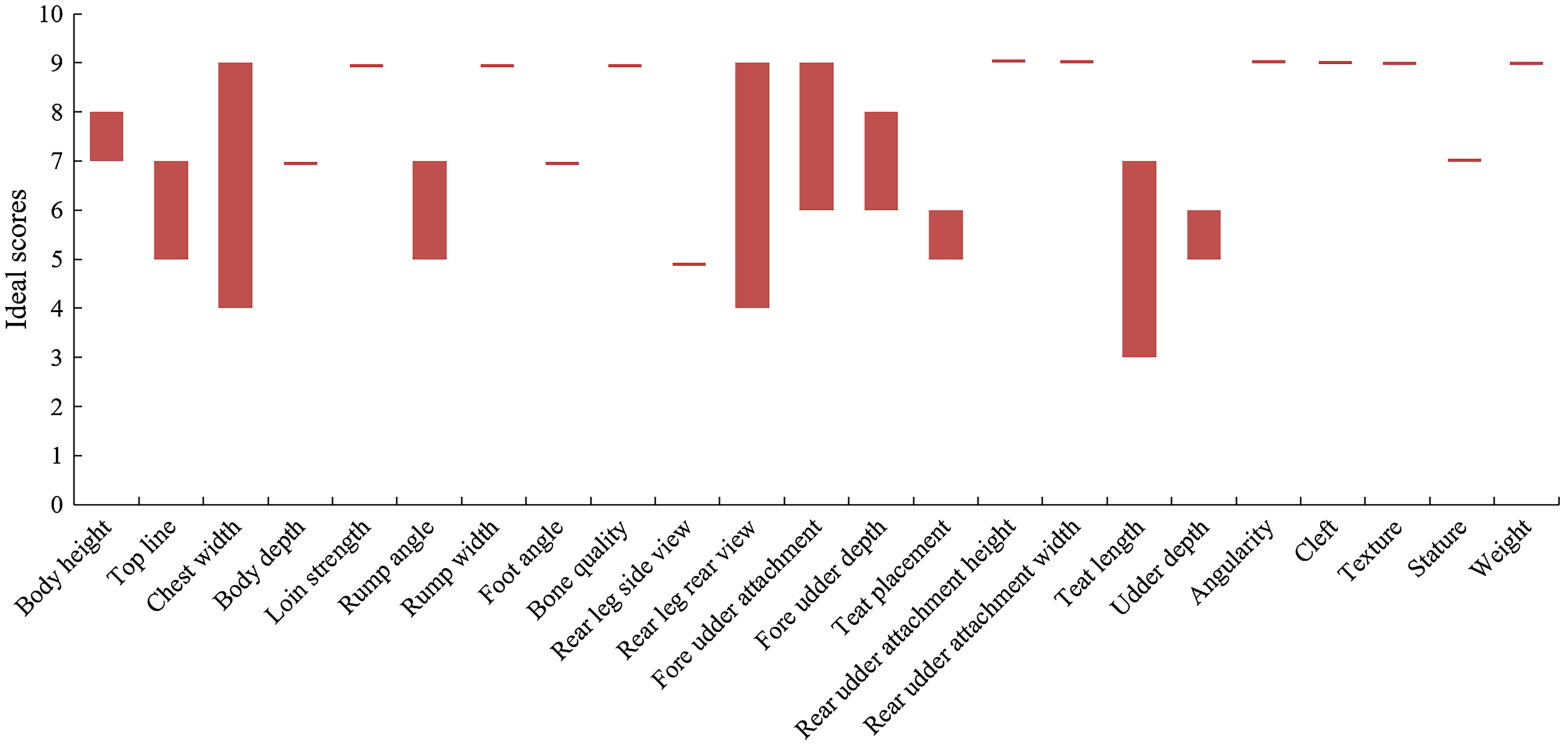
Ideal score for various body traits of dairy cows.

Conformation traits with genetic traits can be monitored in early life (usually the first lactation), thus it is an attractive indirect traits of longevity (Miglior et al., 2017). Conformation traits include lactation system traits (breast texture, udder attachment, udder depth, teat placement, median suspensory, rear breast width, and rear breast height), foot and leg traits (bone quality, rear view of the rear leg, and foot angle), and body traits (weight, body height, and body depth). The higher total conformation score, the longer the longevity. Dairy workers worldwide have been committed to improve the longevity of dairy cows by looking for the ideal score for each conformation trait and exploring the correlation between these traits and longevity (Figure 1; Zavadilova et al., 2009, 2011; Kern et al., 2015; Mao, 2015; Imbayarwo-Chikosi et al., 2016). The nine-point scale is often used to score the conformation traits of dairy cows, and the optimal score value is selected only when the productive life is longest and the milk yield is highest.

**Figure 2:**
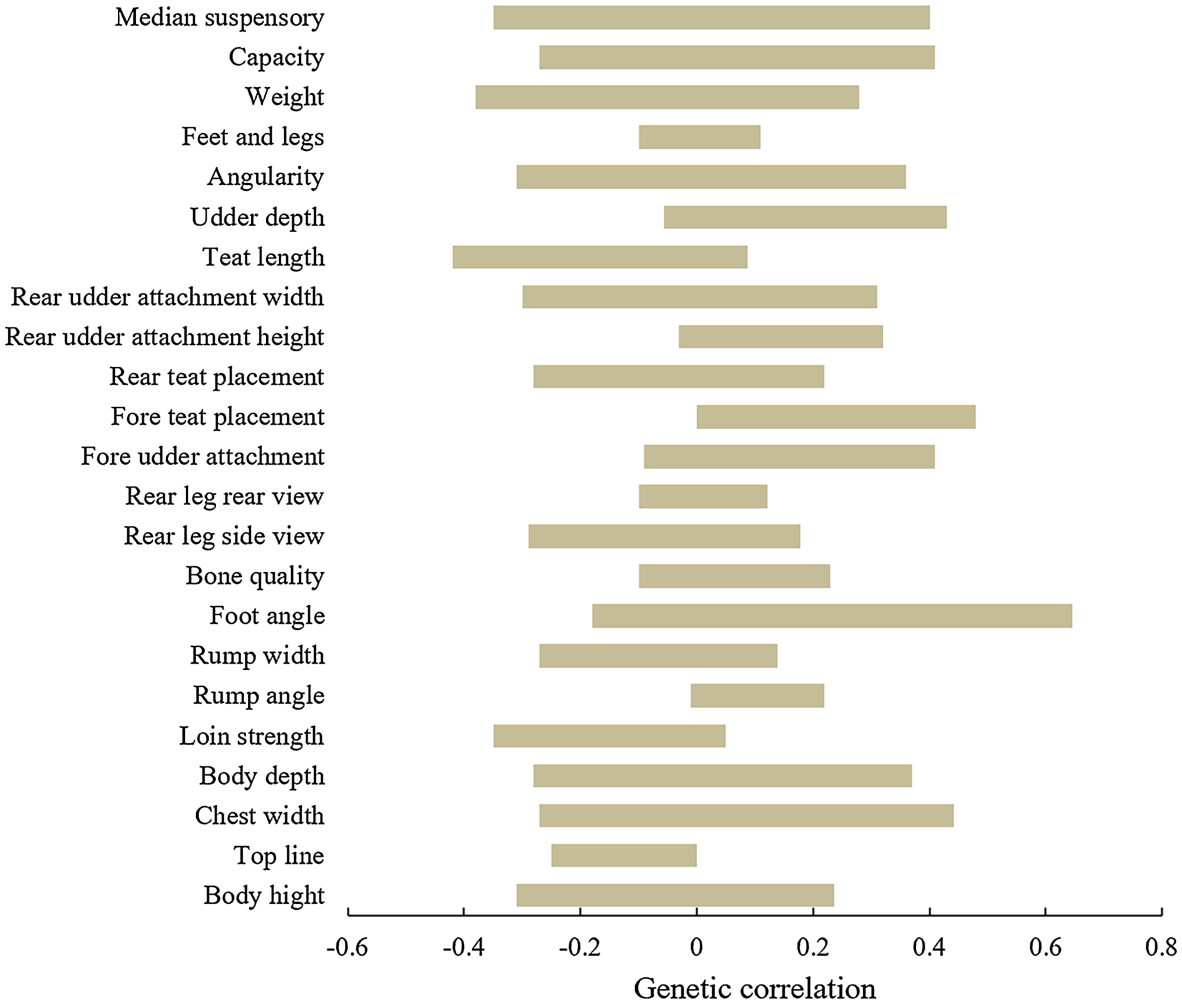
Genetic correlations between longevity and type traits.

Longevity has a weak genetic correlation with the lactation system and body condition (Zavadilová and Stipkova, 2012), and it is unfavorable for selecting the longevity trait. While body height, chest width, loin strength, rump angle, rump width, foot angle, bone quality, rear view of the rear leg, breast texture, fore udder attachment, rear udder attachment, udder depth, teat placement, median suspensory, and top-line were all positive genetic correlated with the longevity (Sewalem et al., 2004; Wu, 2007), and it is favorable for longevity trait selection, among these traits, foot angle and rump width are the highest correlations with the productive life of cows. On the contrary, there is a significantly negative genetic correlation among teat length, fore teat placement, and longevity (Vacek et al., 2006; Čanji et al., 2008). Moreover, several studies have found that other traits are negatively genetic correlated with longevity (Vollema and Groen, 1997; Perez-Cabal et al., 2006; Muntean et al., 2018). Above all, longevity has a high genetic correlation with lactation system traits, a low genetic correlation between foot and leg, and a low or medium negative genetic correction with body traits (Figure 2). These differences among studies may be due to the different breeding needs, herd size, genetic background, the term of longevity, and the analytical models used in the study of cow longevity in different countries. Additionally, the oral score also plays an important role in productive life. Muntean et al. (2018) found that Bolan cows with a better oral score (score of 1) have longer life expectancy. If degeneration of the incisors affects the feeding ability of the cows, which in turn affects the nutritional level needed to maintain physical condition. Cows can live longer and produce more milk when they achieve ideal scores conformation. Therefore, these conformation traits are the ideal indirect trait for selecting and breeding longevity of dairy cows.

### Longevity and Milk Production Traits

To reduce the cost of reserve cows during the breeding process, it is important to select cows with excellent milk production and longevity (Wasana et al., 2015). A study of 36,663 Slovenian brown cattles found that longevity was positively genetic correlated with 305-day milk yield, with a correlation coefficient of 0.23 (Jenko et al., 2015). Dairy cows had a longer lifespan and higher final milk production when the first milk production of primiparous cows was less than 30 L/d (Haworth et al., 2008). Kaupe et al. (2007) studied 1,291 Holstein cows and found that longevity was significantly negatively phenotypic correlated with milk fat and somatic cell count, with a correlation coefficient of −0.08 and −0.52, respectively, and longevity was positively phenotypic correlated with protein content, with a correlation coefficient of 0.01. Longevity had a significant positively genetic correlation with milk fat and milk yeild, the correlation coefficients are 0.46 and 0.43, respectively (Weigel et al., 1998), and it was positively genetic correlated with fat and protein, the correlation coefficients are 0.56–0.61 (Haile-Mariam and Pryce, 2015). However, other studies have found a negative correlation between production life and milk production (Mirhabibi et al., 2018; Bieber et al., 2019), which indicate that high-yield cows have high energy metabolism during lactation, and their breasts may be more vulnerable to the milking equipment. There is a positive genetic correlation between somatic cells and blood immune-related cells. Sartori et al. (2020) found that the high positive genetic correlation between longevity and fighting ability (average ra = 0.669). Therefore, a higher resistance to mastitis is associated with their better welfare and longer the lifespan of dairy cows, which lead to a higher milk yield and production to farm benefit.

Furthermore, the urea nitrogen content, lactose content, and milking temperament also impact longevity of dairy cows. Miglior et al. (2006) found that there was a linear correlation between the lifespan and the urea nitrogen content, cows culling rate decreased with the increasing of urea nitrogen content, but the cows culling rate would increased when the content of urea nitrogen still increased to a certain extent. Because urea nitrogen content above 19 mg/dL had an important influence on the reproductive traits of dairy cows (Wang et al., 2010), which would increase their return to estrus and shorten their production life. On the contrary, the content of urea nitrogen less than 12 mg/dL might reduce milk production and milk protein content by affecting the rumen microflora quantity and reducing dietary protein index, thereby shortening the life of dairy cows (Zhang et al., 2011). Costa et al. (2020) found that the lactose content in milk decreased and dairy cows were more likely to be eliminated with the increase of breast infection degree and parity. Miglior et al. (2006) also found that cows with low lactose level had higher risk of elimination, whereas dairy cows with high lactose levels had a low risk of elimination. Because high lactose level may affect the pregnancy rate, early luteal reaction, and subclinical and clinical ketosis of dairy cows, which can shorten lifespan. Milking temperament can be defined according to milking behavior (Hedlund and Løvlie, 2015) and aggressiveness occurs during feeding (Uetake et al., 2004). Cows with a fast milking speed have a higher somatic cell count and susceptible mastitis (Sewalem et al., 2010). A docile temperament is beneficial for increasing the calving rate, conception rate, milk production, and production life (Chang et al., 2019).

### Longevity and Reproductive Performance

Improvement in reproductive performance and the using of sex-controlled semen increased the number of replacement heifers in most dairy economies, which also cull more reserve cows (Overton and Dhuyvetter, 2020). Sewalem et al. (2005) analyzed the factors affecting the herd life of Holstein, Jersey, and Ayrshire cows, and found that the risk of being culled was higher for older heifers than for heifers calving at an age between 24 and 28 months in all breeds. Late calvings are presumably caused by some problems associated with herd management, fertility, other health problems, and higher rearing costs. In addition, cows first calving at <21 month of age have a high risk of culling due to dystocia and the high elimination is related to the quota system that exists in Canada (Nilforooshan and Edriss, 2004). Anim et al. (2020) analyzed different lactations recordings and found that productive life of dairy cows gradually decrease with the increasing of the age of the first calving, especially for cows calving at an age over 29 months. Mossa and Ireland (2019) found that cows had low fertility, low milk yield, and a high elimination risk with low and high follicle numbers. Therefore, to reduce costs, cows should be bred for the first time at approximately 14 months so that they can achieve the first calving at approximately 23–24 months. However, it is important to choose the first calving time of the cows according to the climate and breeding needs of their country.

The calving interval is a reproductive trait with low heritability (Pryce et al., 2000). The ideal state of dairy cows is lactation for 10 months and dry milk for 2 months. When the time of artificial insemination is less than 60 days, the conception rate is found to be significantly lower. Do et al. (2013) found that there was a negative correlation between the first two calving intervals and the production life of dairy cows (the correlation coefficient is −0.265), and cows had a longer calving interval and higher milk production with a short production life (Wu, 2007). Calving ease significantly reduces the service life and reproductive performance of dairy cows (Maturana et al., 2007). Although the criteria for calving difficulty vary from country to country, the result is that unassisted cows live longer than cows that require assistance during delivery (Hossein-Zadeh, 2016). Dystocia reduces herd profitability, impairs the reproductive performance of cows in the next breeding cycle, and reduces milk production (Hossein-Zadeh, 2016). Rajala-Schultz and Gröhn (1999) found that the risk of culling for dystocic cows was significantly increased during the first 30 days after calving and at the end of the lactation. In addition, the number of pregnancies (Molina-Coto et al., 2020), open days (Pinedo and Vries, 2010), and calf survival rates significantly increased the risk of cows being eliminated (Yalew et al., 2011). Therefore, there was a low or moderate genetic association between reproductive traits and longevity traits, it is necessary to continue to further examine the correlation between these traits and longevity in follow-up studies of the breeding traits of dairy cows. In particular, the first calving, calving interval, calving ease, and calf survival rates may be the indirect traits that can be used for the early selection for longevity.

### Longevity and Disease Traits

There is a unfavorable genetic correlation between longevity and health traits, especially metabolic diseases, which have a strong genetic impact on productive life, and the genetic correlation is −0.98 (Shabalina et al., 2020). Therefore, health traits can be considered as an index to measure productive life. Hadley et al. (2006) found that up to 80% culled dairy cows had the health problems. Therefore, appropriate treatment methods should be adopted to reduce the prevalence of disease in dairy cows to the greatest extent. The main diseases related to the longevity of dairy cows include mastitis, foot disease, metabolic disease, reproductive disease, digestive tract disease, tuberculosis, and brucellosis. Dairy cows suffering from clinical mastitis and reproductive diseases are the most likely to be culled, which have low or moderate negative genetic correlations with longevity (Holtsmark et al., 2008). The culled risk during the remaining lactation period increases after cow suffering from mastitis, and mastitis resistance is positively correlated with productive life (Neerhof et al., 2000). Therefore, longevity can be extended by selecting cows with high resistance to mastitis. Reproductive diseases, such as abnormal estrus, retained placenta, ovarian quiescence, ovarian cysts, persistent corpus luteum, and endometritis abortion can shorten the lifespan, which can prolong the calving interval and decrease milk production (Pascottini and Leblanc, 2020).

Foot disease affects the longevity of dairy cows by affecting their activities, feeding behavior, and production performance. Charfeddine and Perez-Cabal (2016) found that the existence of ulcers or white lines in foot disease was associated with low milk yield of Spanish Holstein cows, the existence of ulcers, or white line disease in early lactation will extend day open and long calving intervals. Metabolic disease is an important cause for the involuntary culling of dairy cows. Milk fever is the most important disease affecting the survival rate, followed by ketosis, fatty liver, and abomasal translocation (Probo et al., 2018). These diseases are related to both longevity and milk production, the risk of dairy cow culling increases when one metabolic disease coexists with another. More importantly, the metabolic stress caused by metabolic diseases can damage the mitochondria and further affect the longevity of dairy cows. Ketosis is the most important metabolic disease in all countries (Huber et al., 2016); ketosis will secrete less milk and milk fat content. The sick cow will no longer continue to secrete milk with the aggravation of the disease, consequently, they will be involuntary culled and the production life will be shortened.

Bovine infectious tuberculosis and brucellosis are two major infectious diseases in dairy cattle breeding, which not only endanger human health but also shorten the longevity of dairy cows (Ma and Xie, 2020). Breast tuberculosis is an infection of the mammary gland that results in a lump on the outside of the breast, causing in low milk production and short lifespan of cows (Yang and Zhou, 2010). Brucellosis is a common epidemic disease in pastures; cows suffering from brucellosis are prone to abortion, retained placenta, and long-term infertility (Wang, 2020). Additionally, the longevity traits of dairy cows in various countries with the increasing understand, the longevity of dairy cows should not only be studied after calving, but also before calving. The calf survival rate is affected by respiratory disease and diarrhea, which occur annually in most intensive dairy cows (Timsit et al., 2017). According to the National Animal Health Surveillance System of the United States, half of the deaths of calves in the United States dairy industry are caused by diarrhea (Yong-II and Kyoung-Jin, 2014). Therefore, a cow herd with high disease resistance will be beneficial to extend the longevity of the cows.

### Longevity and Nutrition

Nutritional factors are directly or indirectly related to the growth and development of animals, physiological and biochemical indices, and immune indices (Michael et al., 2019). High protein levels can have various toxic effects on the ovum, oosperm, and embryo, and it will reduce prostaglandin synthesis and progesterone, delay estrus, and ovulation. Moreover, low protein content in the diet will cause reproductive diseases, such as delayed follicular development, abnormal estrus, low conception rate, and retained placenta (Liu, 2017). Excessive carbohydrate content in the diet will lead to rumen acidosis in dairy cows (Agovino, 2018). An imbalance in the calcium and phosphorus ratio can readily cause the development of metabolic disorders and osteoporosis. The feeding, ruminating, and digestion characteristics of cows are significantly different at different ages. Therefore, total mixed ration (TMR) technology should be promoted according to the nutritional needs of dairy cows at different ages, growth stages, pregnancies, and lactation (Schingoethe, 2017). The roughage and concentrate should be cut, stirred, mixed, and fed in proportion to ensure a balanced nutritional intake for dairy cows. Additionally, regular sampling and analysis of the TMR mixed diet should be carried out to reduce the inconsistency between the trough feed and the allocated feed in the mixed diet, because imbalance nutrient can result in diseases and thereby shorten the longevity of dairy cows.

### Longevity and Management

The improvement of cow comfort and welfare is conducive to improve the profitability of dairy farms and longevity. Bouffard et al. (2017) studied Holstein cows in 100 tiestall dairy farms and found that most cows tied in the cattle pen had an increase in the incidence of foot disease and shortened the lifespan by comparison free-ranging of cows. Fuerst-Waltl et al. (2018) found that the culling rate of free-ranging Simmental cattle was 15% lower than stall-fed Simmental cattle. Exercise may be an important factor for improving the health of dairy cows. Therefore, it is important to adhere to the concept of animal welfare and provide more consultation services for the breeding of dairy cows (Karin et al., 2018). It is important to control the herd size (Sawa et al., 2016), keep warm in winter, and provide sufficient ventilation to prevent heatstroke in summer. In addition, flooring type and slipperiness, barn cleanliness, bedding type and quantity, and stall design are all associated with increased odds of lameness (Wu et al., 2018). Additionally, managers should regularly assess performance and health status data in accordance with the range management system to detect adverse trends for timely treatment. In daily life, cows should have the opportunity to bask in the sun every day, sufficiently brush the cow’s body, which ensures they are clean and dry at all times to promote blood circulation and metabolism and grasp the suitable age for breeding. The breasts should be massaged every day, which maintains normal function of breast and promotes lactation. Ensuring cows should have appropriate daily activity to improve their physique, feed conversion time, and efficiency, which in turn ultimately improve the milk yield. In particular, the dirt between cows toes should be cleaned regularly, and the cows’ feet should be pruned. Protecting the cows’ feet in strict accordance with the operating procedures of foot repair technology will reduce the occurrence of limb foot disease (He, 2011).

## Longevity in Breeding Systems in Various Countries

For many years, most selection indices worldwide focused on increasing milk production (Miglior et al., 2005). With relative emphasis on production in various countries, the selection indices have gradually shifted toward a more balanced breeding goal of improving production, especially protein yield and percentage, longevity, udder health, conformation, and reproduction (Vanraden, 2002). Longevity of dairy cows has been studied in many countries since the 1950s (Zhang et al., 2020). Longevity traits are heritable and can be improved by selecting (Miglior et al., 2017). The definition and model of longevity traits are also developing with further research on longevity traits in developed countries (Miglior et al., 2005). However, there is currently no consensus on definition of longevity trait or on the methodology for the evaluation across countries. Therefore, the definition and model for longevity trait selection, and the longevity trait weight in the comprehensive selection index of each country is inconsistent.

The United States considers productive life, which combines direct longevity defined as total months in milk through 84 mo of age, along with somatic cell score, milk yield, milk fat yield, milk protein yield, and some conformation traits (Cruickshank et al., 2002), and usually analyzed by single trait-best linear unbiased prediction (BLUP)-animal model. Canada considers longevity is a total five traits, survival status from the first calving to day 120, day 120 to 240, day 240 to second calving, second to third calving, and third to fourth calving, which combines direct longevity somatic cell score, milking speed, non-return rate, calving to first service interval, and some conformation traits, and usually analyzed by five traits-BLUP-animal model (Sewalem et al., 2007). Nodic considers longevity is a total of five traits, the partial productive life from the first to the second, third, fourth, fifth, and sixth calvings, which usually analyzed by muti-traits-BLUP-animal model. Germany considers longevity is a total of nine traits, survival status from day 0 to 49, day 50 to 249, and day 250 to the next calving after the current calving among the first three lactations, which usually analyzed by muti-traits -BLUP -animal model (Zhang et al., 2020). France considers productive life, which combines direct longevity along with somatic cell count, clinical mastitis, and some reproduction and conformation traits, which usually analyzed by Single trait-survival analysis–Sire-maternal grand sire model (Zhang et al., 2020). Australia considers longevity is survival status from the first to the second, third, fourth, fifth, sixth, seventh, and eighth calvings, which usually analyzed by muti-traits-BLUP-animal model (Zhang et al., 2020).

Longevity was included in the total performance index (TPI) by the American Holstein Dairy Association in 2001, which accounted for 8% of the latest TPI established in 2021. Canada added longevity to the lifetime performance index (LPI) in August 2001, longevity accounted for 5% of the latest LPI index in 2021. In 1990, longevity is added to the nordic total merit (NTM), and longevity accounted for 7% of this index. In Germany, milk yield, conformation, and functional traits were first included in the selection index relative zuchtwert gesamt (RZG) in 1997, function longevity accounted for 20% of the latest RZG index in 2020. Longevity accounted for 5% of the French Index de Synthèse UPRAISU in 2021. The weight of longevity traits in the TPI of Australian and Dutch dairy cows was 8 and 12%, respectively. Therefore, longevity plays an increasingly important role in dairy cattle breeding in various countries.

## Breeding and Genetic Selection

Improvements and genetic selection for milk yield have led to substantially steady increase in milk production over recent decades in many countries, including Canada, the United States, and throughout Europe and Australasia (Schuster et al., 2020). However, due to only focus on the improvement of milk yield, other traits, and the disease resistance of cows decrease (Ingvartsen et al., 2003). Therefore, the aim of breeding in various countries gradually shift to more balanced breeding, functional traits, and especially longevity traits. All functional traits have also been included in the selection indices of respective countries (Miglior et al., 2005). There are two approaches to study longevity, the first is to select longevity directly, and the second is to select the underlying functional traits as the breeding goal. The indirectly selects for traits that are difficult to measure or do not have complete data records (Vollema, 1998), and it is important to determine the heritabilities and correlations of these traits.

Since longevity is a low-heritability trait globally (Schuster et al., 2020), and true longevity of cows cannot be known until cows were culled, which makes a longest generation interval (Ducrocq et al., 1988). The emergence of genomic selection technology has shortened the generation interval, improved the breeding value, and accelerated the genetic progress of longevity (Liu et al., 2011). Although genomic selection accelerates genetic improvement, cows can be replaced slightly faster, and raise all born heifer calves to replace cows is not the most profitable strategy in many cases (Vollema, 1998). Thus, the optimal asset replacement theory is only the necessary number of heifers is selected and the lowest genetic merit is sold, which can help to expand the dominant population (Weigel et al., 2012).

In addition, the underlying molecular mechanisms of longevity remain not to be incompletely understand, which slow research progress on the longevity of dairy cows. Molecular breeding methods can more accurately determine the genetic potential for specific traits. At present, many molecular markers, genotypes and metabolites are being assessed in terms of their correlations with the longevity of dairy cows, which can assist in the selection of longevity traits in dairy cows.

**Figure 3:**
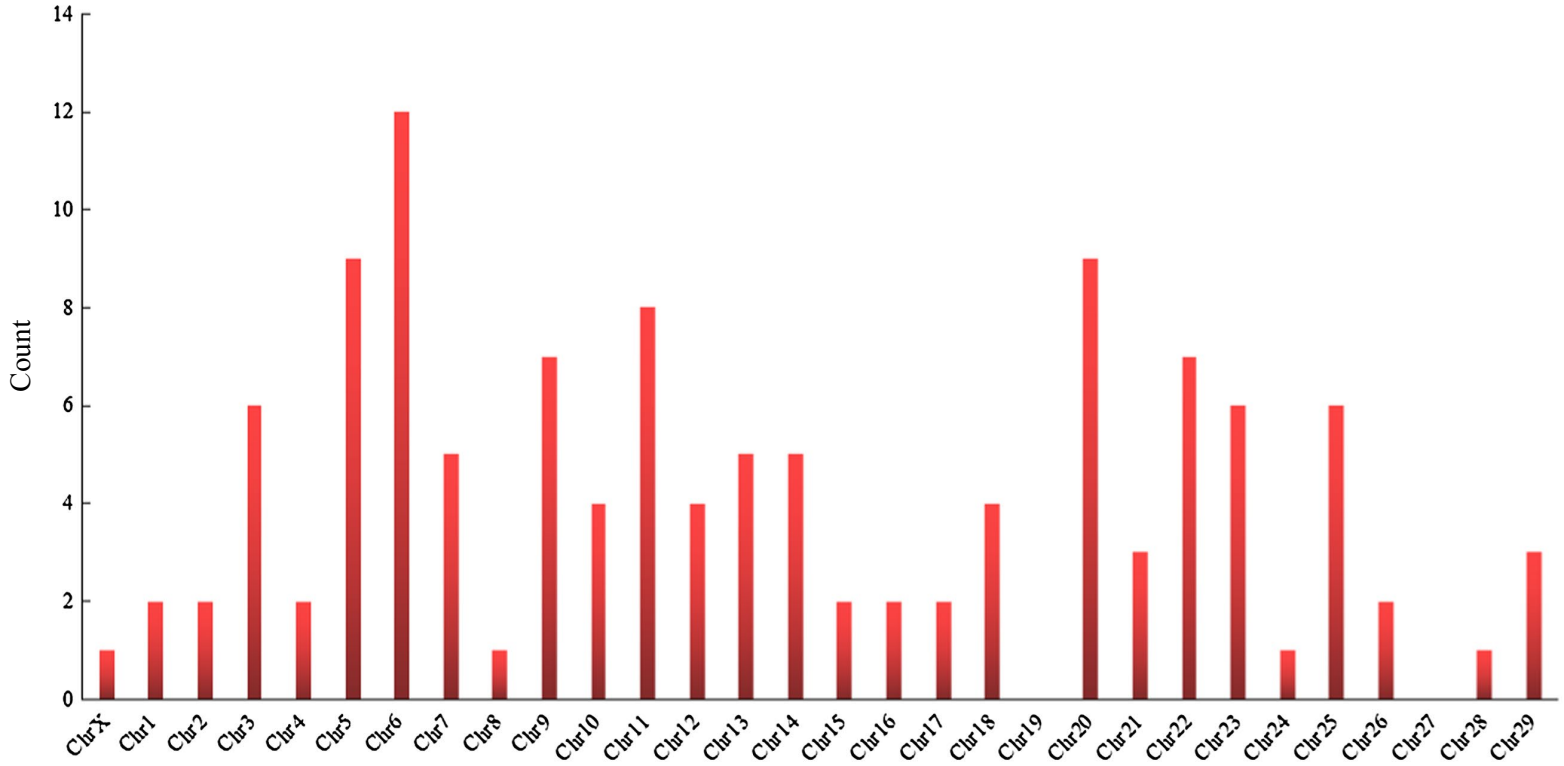
Chromosomal distribution of genes associated with productive life.

**Figure 4:**
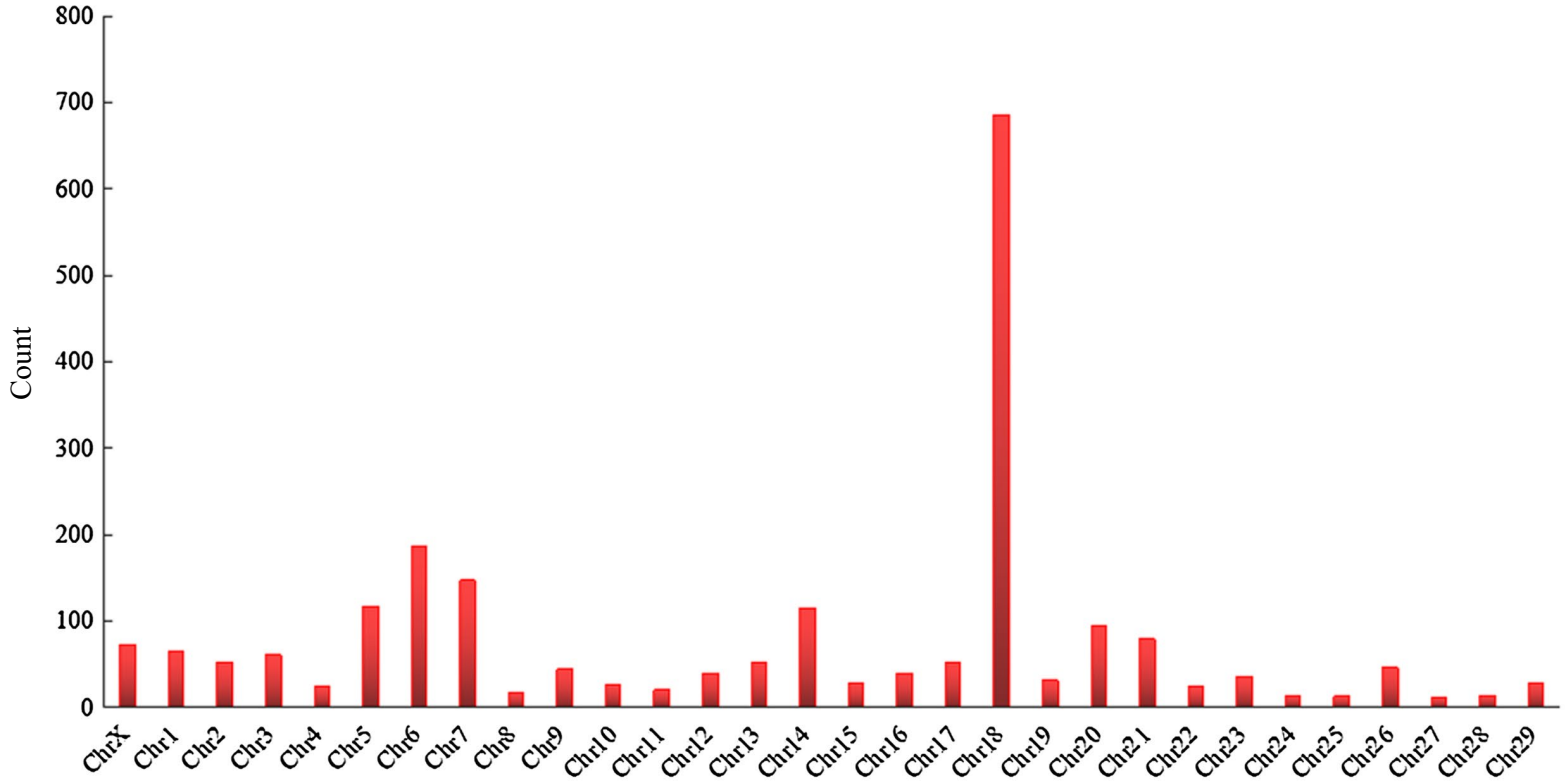
Chromosomal distribution of QTL associated with productive life.

Seeker et al. (2018) found that bovine relative leukocyte telomere length (RLTL) was a heritable trait, and the association with longevity traits can be used in breeding programs to increase the lifespan of dairy cows. Bovine telomeres shorten with age, and the relationship between RLTL at different life stages and the productive life of dairy cows has not been explored. RLTL is generally positively correlated with longevity in humans and vertebrates, but telomere length is negatively correlated with longevity, which implies that short telomeres are associated with an increased mortality risk (Wilbourn et al., 2018). The only other study on the relationship between RLTL and productive life displays a significant but weak correlation in dairy cows (Brown et al., 2012). Therefore, a follow-up study is needed to assess the longevity benefits of a strategy of breeding according to RLTL through genome-wide association analysis. Huber et al. (2016) sequenced 19 dairy cows plasma by using a metabolomics approach to identify the metabolites of long-chain acylcarnitine, spermidine, and biogenic amines associated with prolonged production life, but these metabolites have not yet been confirmed. If these potential new biomarkers are confirmed, they can be used for the genetic selection of bulls and dairy cattle breeding, and increase the number of dairy cows with “extended life” metabolic genetic traits. Ioannidis et al. (2018)found that plasma microRNAs were associated with telomere length, milk yield, milk composition, somatic cell count, reproduction, and blood metabolites related to body energy balance and metabolic stress, suggesting that these microRNAs may be significantly associated with a productive life. Additionally, researchers from various countries have used genome-wide association studies (GWAS) and single-nucleotide polymorphism (SNP) to identify many genes, quantitative trait loci (QTLs), and SNPs are significantly related to longevity (Table 3), indeed, a large number of genes (Figure 3) and QTL (Figure 4) have been discovered from cattle QTL projects.^1^ At present, these genes and polymorphisms are also being studied and tested to determine their relationship with longevity and extend longevity of dairy cows.

**Table 3:** Genes, single-nucleotide polymorphisms (SNPs), and quantitative trait loci (QTL) related longevity of cattle.

Author	Country	Breed	Definition	Analytical method	chromosome	Gene, SNP, and QTL
Pryce et al., 2010	Australia	Holstein, Jersey	Longevity	SNP-by-SNP	6, 26	13.5–23.7 Mb (6 SNP), 70.4–75.6 Mb(5 SNP), and 0.33–1.46 Mb(6 SNP)
Ashwell and Tassel, 1999	United States	Holstein	Productive life	Microsatellite Marker	2, 21, 23	BMS2519, BM103, and 513
Saowaphak et al., 2017	Thailand	Holstein	Productive life	GWAS	5, BTAX	SYT1, DOCK11, IL13RA1, and KLHL13
Nayeri et al., 2017	Canada	Holstein	Longevity	GWAS	18, 6, 14	CTU1, NPFFR2, CYHR1, CPSF1, DGAT1, and GRINA
Zhao, 2013	China	Holstein	Productive life	SSCP	12	FoxO1(TT)
Wang et al., 2018	China	Holstein	Productive life	SNP mutation analysis	2, 3	CXCR1-816 A>C, LF-28A>C, and SLC35A3-66G>T
Steri et al., 2019	Germany	Holstein	Longevity	GWAS	16, 30	43.25–43.69 Mb, 17.56–18.06 Mb
Szyda et al., 2011	Poland	Holstein	Functional longevity	SNP mutation analysis	3, 4, 6, 14, 23	LEPR, LEP, ABCG2, DGAT1, and BTN1A1
Bjelka and Novák, 2019	Czech Republic	Czech Red Pied	Longevity	HiSeq X-Ten	6	TLR1 798C>T, TLR1 1762G>A
Khatib et al., 2005	United States	Holstein	Productive life	SNP mutation analysis	21	PI
Tassell et al., 2000	United States	Holstein	Productive life	Microsatellite Markers	2, 21, 23	BMS2519, BM103, and MB026
Mészáros et al., 2014	Sweden	Fleckvieh	Longevity	GWAS	5, 6, 8, 14	SYT10, ADAMTS3, NTRK2, and SNTG1
Cole et al., 2011	United States	Holstein	Productive life	GWAS	7, BTAX	INSR, LOC520057, and GRIA3
Steri et al., 2019	Italy	Holstein	Longevity	GWAS	16, 30,	UBIAD1, MTOR, ANGPTL7, EXOSC10, SRM, MASP2, TARDBP, CASZ1, GPC3, and PHF6
Zhang et al., 2016	Netherland	Holstein, Red Dairy Cattle, Jersey	Longevity	GWAS	23, 9, 10	KCNK16, PPP1R14C, and GCH1
Kaupe et al., 2007	Germany	German Holstein	productive lifespan	SNP mutation analysis	21	UTMP SNP1296
Zhang et al., 2021b	China	Chinese Holsteins	Productive life; Herd life; milking life; Partial Lifespan	GWAS	2, X, 9, 18, 4, 20, 25	RPRM, GRIA3, GTF2H5, CA5A, CACNA2D1, FGF10, and DNAJA3
Shabalina et al., 2020	Germany	Holstein	Length of productive life	GWAS	4, 10, 13, 28	ETV1, ONECUT1, MACROD2, and SIRT1

## Conclusion and Future Perspectives

The dairy industry is gradually moving toward intensive, large-scale, standardized, and mechanized breeding worldwide. With the increasing attention being paid to the breeding value, lifetime benefits, climate change, and environmental sustainability of dairy cattle globally, the focus of dairy cattle breeding has begun to shift to more balanced breeding goals, including longevity, health, welfare, milk yield, milk quality, and environmental sustainability. Longevity have been incorporated into breeding programs in many developed countries according to the dairy production importance (Miglior et al., 2005), and have been selected for longevity traits. Extending of dairy cow longevity has become an urgent need for the development of the dairy industry. Nevertheless, longevity does not yet include into national selection indices in many developing countries because of the complexity. For example, the newly revised Chinese Dairy Performance Index (CPI) in 2020 only includes milk protein content, milk fat content, somatic cells, conformation, lactation system, and feet, and longevity has not been included in the selection index. To increase the breeding of longevity traits, it should be included in selection indices in each country, because longevity trait has economic value as its improvement that can reduce production costs (Allaire, 1981). It is worth noting that the correlation between longevity and milk yield remains unclear. In the future, if longevity is included in the selection index, it will be necessary to comprehensively consider whether it will affect the milk yield of dairy cows.

The short longevity of dairy cows not only seriously affects productivity, but also hinders the scope for selection for other traits. In traditional breeding, researchers in various countries have used different models and trait definitions to directly or indirectly select for longevity traits (Gill and Allaire, 1976; Hoque and Hodges, 1980; Buenger et al., 2001). Multiple-trait evaluations combining indirect measures of longevity with direct measures are helpful to improve the accuracy of longevity evaluations (Miglior et al., 2017). In addition, the terms used to describe the longevity trait are inconsistent across countries, and if all terms are used interchangeably, there will be inconsistencies and ambiguities in the definitions of longevity (Caraviello et al., 2004; Brickell and Wathes, 2011; Raguz et al., 2011). Therefore, it is necessary to standardize the different terms related to longevity traits, and researchers can choose different definitions of longevity depending on the purpose of their study.

With the development of molecular technology, genome selection can significantly improve the genetic improvement speed of dairy cows and shorten the generation interval. However, genomic selection accelerates herd replacement, the accuracy gradually decreases due to recombination and the linkage between SNPs and causal genes disappears with time (Zhang et al., 2021a). Longevity traits have low heritability and are highly influenced by the environment, since dairy cattle are farmed globally, and the environment varies greatly from country to country, so it is important to study the interaction between longevity traits and the environment. In the future, it will be particularly important to correctly identify the early indicator traits and genetic markers of longevity, improve the accuracy of longevity assessment, collect complete records of dairy cows’ conformation traits, lactation system, reproductive traits, health traits, limbs, and hooves, and perform dairy herd improvement (DHI) determinations. However, it is important to find a balance between collecting record data and resulting benefits to farmers. Good performance recording in combination with an appropriate model of genetic evaluation and a well-organized selection process have been shown to be useful for breeding of longevity in dairy cows, which will make better progress in this field. In additional, national policies and animal welfare can be a challenge in choosing a longevity herd or the optimal herd in breeding longevity traits in the future when production efficiency and profit are the primary goals. Therefore, the future selection of dairy cattle for longevity breeding will require a fully integrated and balanced breeding model.

## Author Contributions

HH and TM contributed to the writing, preparing the original draft, investigation and validation. YaM and XW contributed to revision and supervision. YuM contributed to the conceptualization. All authors contributed to the article and approved the submitted version.

## Conflict of Interest

The authors declare that the research was conducted in the absence of any commercial or financial relationships that could be construed as a potential conflict of interest.

## Publisher’s Note

All claims expressed in this article are solely those of the authors and do not necessarily represent those of their affiliated organizations, or those of the publisher, the editors and the reviewers. Any product that may be evaluated in this article, or claim that may be made by its manufacturer, is not guaranteed or endorsed by the publisher.

## Footnotes

^1^ https://www.animalgenome.org/cgi-bin/QTLdb/index
